# A Methodology to Quantify Resilience in Growing Pigs

**DOI:** 10.3390/ani11102970

**Published:** 2021-10-15

**Authors:** Houda Laghouaouta, Ramona N. Pena, Roger Ros-Freixedes, Josep Reixach, Marta Díaz, Joan Estany, Ramon Armengol, Anna Bassols, Lorenzo Fraile

**Affiliations:** 1 Agrotecnio-CERCA Center, Department of Animal Science, University of Lleida, 25198 Lleida, Spain; houda.laghouaouta@udl.cat (H.L.); romi.pena@udl.cat (R.N.P.); roger.ros@udl.cat (R.R.-F.); joan.estany@udl.cat (J.E.); ramon.armengol@udl.cat (R.A.); 2 Selección Batallé SA, 17421 Riudarenes, Spain; jreixach@batalle.com (J.R.); mdiaz@batalle.com (M.D.); 3 Departament de Bioquímica, Universitat Autónoma de Barcelona, 08193 Barcelona, Spain; anna.bassols@uab.cat

**Keywords:** body weight, haptoglobin, pigs, resilience indicators, vaccine challenge

## Abstract

**Simple Summary:**

The concept of resilience can be defined as the ability of animals to maintain their productivity despite the stressors that might occur during the rearing period. Despite the growing interest in the genetic determinism of resilience and its possible inclusion in selection objectives, there is no straightforward method to measure this trait. Several quantifying methods estimate resilience based on productivity such as body weight or milk production, or non-productivity related traits such as immunity or stress responses. The objective of this study was to elaborate novel resilience indicators in growing pigs based on both productivity (body weight) and non-productivity (acute-phase proteins) related traits. We propose the deviation from the expected growth curve and the increment of the acute-phase protein haptoglobin, after applying a common vaccine, as resilience indicators in growing pigs under standard rearing conditions. We showed that the suggested resilience indicators are under a genetic control, show a substantial variability in the population, and may be improved through selection.

**Abstract:**

There is a growing concern about the genetic determinism of resilience and its possible implementation in breeding programs. The objective of our study was to elaborate novel resilience indicators in growing pigs based on the deviation from the expected growth curve and the increment of the acute-phase protein haptoglobin (HP) after applying a common vaccine. A total of 445 pigs were vaccinated with an attenuated Aujeszky vaccine at 12 weeks of age. Deviation from the expected body weight (ΔBW) given the growth curve of unvaccinated pigs at 28 days post-vaccination (DPV) and the increment of HP at 4 DPV (ΔHP) were suggested as resilience indicators. Challenged pigs that maintained their productivity and had a minor activation of HP were deemed resilient, whereas pigs that had low ∆BW values and a high activation of HP were deemed susceptible. Pigs were also classified based on ∆BW and ∆HP relative to the expected BW at 28 DPV and to the basal level of HP, respectively. The concordance was high between both methods, indicating that ΔBW and ΔHP are not sensitive to the animal’s expected BW nor the basal level of HP. The heritability estimates were moderate for ∆BW (0.33) and low-to-moderate for ∆HP (0.16). Our study suggests ΔBW and ΔHP as novel resilience indicators in pigs. The suggested indicators capture different aspects of resilience, are easy to measure, and are genetically controlled. Thus, they may be improved through selective breeding. Further analyses are needed to validate our findings.

## 1. Introduction

Pork is one of the most important sources of animal protein for humans and a sustained increase in pig production will be necessary to cope with the challenge of providing worldwide food security [1]. This challenge can be tackled by increasing the number of animals and by improving the efficiency of the pork production sector. Each animal is exposed to a diversity of stimuli that arise from its internal and external environments and it is desirable for the animal’s welfare and its commercial productivity, that it, has the capacity to cope with these challenges. This defines the concept of resilience, that is, the ability of the animal to be minimally affected by all the perturbations that might occur during its productive life and quickly return to the physiological, behavioral, cognitive, health, affective, and production states that pertained before exposure to a disturbance [2]. In recent years, there has been a growing interest in the genetic determinism of resilience and its possible inclusion in selection objectives. Selective breeding for improved resilience would provide animals with more robust phenotypes. However, there is no straightforward quantification method for resilience. Indirect indicators that reflect the disturbance caused by stressors such as residual variance or variation coefficients have been used as proxies to phenotype this complex trait [3].

Although there is a general agreement that internal and external challenges negatively impact productivity through the dysregulation of basic biological mechanisms [4], there is no consensus on how to measure resilience. Several quantification methods estimate resilience based on productivity-related traits such as body weight (BW) in layer chickens [5], feed intake in pigs [6], litter size in rabbits [7], and milk yield in cattle [8,9]. All these methods assume that resilient animals are able to maintain their productivity despite stressors.

The relationship between resilience and immunity has also been well documented in the literature. Stressors modulate the immune system [10,11] and increase disease susceptibility [12]. Resilient and susceptible animals respond differently toward stressors and express different immune phenotypes [10]. In this sense, several resilience indicators have been assessed through non-productivity related traits such as medication records [13] or antibody levels [14]. In this context, acute-phase proteins are frequently measured in relation to the innate immune response. These are a group of blood proteins that change their concentration in animals subjected to infection, inflammation, surgical trauma, or stress [15]. During the first days after the challenge, there is an increase in the plasma concentration of positive acute-phase proteins such as haptoglobin (HP) and a decrease in negative acute-phase proteins. Serum acute-phase protein levels have been used as biomarkers for the presence and extent of disease processes [16]. Given the strong relationship between immune state, welfare, and productivity, lower serum acute-phase proteins have been correlated with better production parameters in pigs [17,18] and therefore could be used as indicators of resilience.

The objective of the present study was to evaluate novel resilience indicators in growing pigs based on both the deviation from the expected growth curve and the increment of the acute-phase protein HP after applying a common vaccine under standard rearing conditions, with the goal of evaluating the capacity of these indicators for classifying animals as resilient or susceptible.

## 2. Materials and Methods

### 2.1. Animals and Experimental Design

Animals belonged to a commercial Duroc line reared in high-health status farms from Selección Batallé SA (Riudarenes, Northeast of Spain). A total of 540 barrows at approximately 10 weeks of age (71.4 ± 2.4 days) were identified, ear-tagged, and randomly distributed into five fattening batches, ensuring a stock density of 1 m^2^ per animal. Pigs were reared under the same conditions and fed ad libitum with commercial diets. After 14 days (85.6 ± 2.4 days of age), 445 pigs (experimental group, E) were intramuscularly vaccinated with an attenuated Aujeszky vaccine (Auskipra, batch 9R83, Laboratorios Hipra, Amer, Girona) and 95 pigs (control group, C) were inoculated with phosphate-buffered saline. Live attenuated Aujeszky vaccines are widely used in swine medicine preventive programs across Europe due to the risk of transmission of wild-type Aujeszky virus from wild boar to domestic pigs. Thus, many animals are routinely vaccinated across Europe with this vaccine and the measurement of haptoglobin, following this vaccination, could be easily carried out without adding any additional vaccine to the pig population. Each rearing batch contained E and C pigs, and half-sibs were evenly distributed between the E and C groups. There is no risk of transmissibility of the Aujeszky virus between E and C pigs due to the well-known safety profile of the vaccine [19]. Experimental individuals were offspring from 49 sires and 198 dams (around 14 sires and 40 dams per batch).

**Figure 1:**
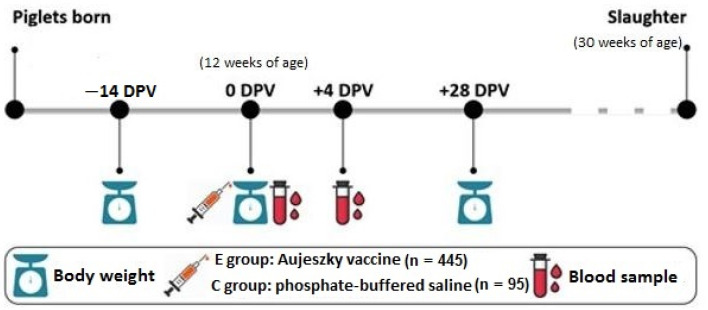
Timeline and experimental design.

Body weight was registered at −14, 0, and 28 days post-vaccination (DPV) and blood samples were collected from the jugular vein at 4 DPV (Figure 1). In addition, 81 pigs (40 from the C group and 41 from the E group) were bled at 0 DPV to establish the basal level of the acute-phase protein HP in each batch. Average daily gain (ADG) between −14 and 28 DPV was estimated. At the end of the fattening period, animals were slaughtered at approximately 30 weeks of age (217.8 ± 3.7 days) following the standard procedures of the production company.

### 2.2. Determination of the Acute-Phase Protein Haptoglobin

Serum HP was quantified at 0 and 4 DPV by using a spectrophotometric method (hemoglobin binding assay) with the Tridelta PHASE Haptoglobin Assay (Tridelta Development Ltd., County Kildare, Ireland) and performed on an automated analyzer (Olympus AU400, Hamburg, Germany). Intra-assay and inter-assay coefficients of variation for this technique have been previously reported [20].

### 2.3. Proposal of Resilience Indicators

The experiment was carried out in commercial farms under standard rearing conditions. Both BW and HP data were analyzed to quantify the pigs’ resilience.

#### 2.3.1. Body Weight Deviation

BW data from the C group were analyzed using the following model:

$${BW}_{28}=B+\alpha {BW}_{-14}+\beta {BW}_{0}+\gamma A+e$$

where BW_28_ is the BW at 28 DPV; B is the batch fixed effect with five levels; BW_−14_, BW_0_, and A are the covariates of BW at −14 DPV, BW at 0 DPV, and age at 28 DPV, α, β, and γ are the regression coefficients; and e is the residual term.

For each pig from the E group, BW_28_ was estimated using the previous model (R^2^ = 85%). This was considered the expected BW_28_ given the growth curve of control pigs. A differential (ΔBW) was calculated as the difference between the observed and expected BW_28_ (ΔBW = observed BW_28_ − expected BW_28_). Besides, %BW was also estimated as the ratio between ∆BW and the expected BW_28_ to have a relative measure of ∆BW. We defined resilient pigs as those minimally affected by the vaccination process and all the uncontrolled events that could have also occurred during the experiment. Resilient pigs were those that had high values of ∆BW and %BW.

Besides, for each pig from the E group, BW_28_ was also estimated based on the pig’s ADG before the vaccine challenge as:

$${BW}_{28}={BW}_{0}+\frac{{BW}_{0}-{BW}_{-14}}{d_{-14,0}}d_{0,28}$$

where $\frac{{BW}_{0}-{BW}_{-14}}{d_{-14,0}}$ is the pig’s ADG before the vaccine challenge (between −14 and 0 DPV) and d_0,28_ is the number of days between 0 and 28 DPV. For each pig from the E group, a differential ΔBW_ADG_ was calculated as the difference between its observed and expected BW_28_.

#### 2.3.2. Increment of Haptoglobin after the Vaccine Challenge

Data of the acute-phase protein HP were analyzed within each batch. A previous work reported that among the five acute-phase proteins (C-reactive protein, serum amyloid A, HP, pig-MAP, and albumin), HP is the most sensitive acute-phase protein for Aujeszky disease [21] and that the peak of its response occurs four days after the infection [22]. Therefore, the increment of HP at 4 DPV (∆HP) was calculated as the difference between the pig’s HP concentration at 4 DPV and the mean of the HP concentration of the corresponding batch at 0 DPV. In addition, %HP was calculated as the ratio between ∆HP and the basal level of HP within the batch. As the plasma concentration of HP increases after the challenge [15], resilient pigs should show lower values of ∆HP and %HP than susceptible pigs.

### 2.4. Classification of Pigs into Resilient and Susceptible Groups

Pigs from the E group were classified as resilient (R), average (A), or susceptible (S) based on BW and HP data. Four different classifications were performed.

1. Pigs were classified based on the combination of resilience indicators ∆BW and ∆HP as suggested by Bai et al. [23]. Pigs with ∆BW above the third quartile (Q_3_) and ∆HP below the first quartile (Q_1_) were classified as R. In contrast, pigs with ∆BW below Q_1_ and ∆HP above Q_3_ were classified as S. The rest of the animals were considered as A.
2. Pigs were classified based on %BW and %HP following the same previous quartile distribution to examine whether ∆BW and ∆HP are sensitive to the animal’s expected BW and the basal level of HP.
3. Pigs were classified based on ∆BW_ADG_ and ∆HP following the same previous quartile distribution.
4. Pigs were classified based on the observed BW_28_ to examine whether ∆BW and ∆HP captured more information than the differences in the observed BW_28_.

The agreement between the different classifications was assessed using a concordance test with the kappa statistic [24]. Correlation between the suggested indicators was assessed using the R software.

### 2.5. Heritability Estimates

Heritability estimates of the suggested resilience indicators (∆BW, %BW, ∆HP, and %HP) were estimated using the following univariate animal model:

y = Xb + Za + e

where y stands for ∆BW, %BW, ∆HP, and %HP; b is the vector of the batch fixed effect and the covariate of age at 28 DPV; a is the vector of additive effects; X and Z are incidence matrices; and e is the vector of residual terms. Additive genetic effects (a) and residuals (e) were assumed to be normally distributed as a ⁓ N (0, Aσ_a_^2^) and e ⁓ N (0, Iσ_e_^2^), where A is the relationship matrix calculated with a five-generation pedigree of 3467 pigs, σ_a_^2^ is the genetic additive variance, I is an identity matrix, and σ_e_^2^ is the residual variance. All effects were assumed to be independent between them. Marginal posterior distributions were estimated using Monte Carlo Markov chains of 1,000,000 samples with a burn-in period of 200,000, of which one in every 100 samples was retained. The heritability was estimated as the mean of the marginal posterior distribution. The probability (P_0.10_) of h^2^ being greater than 0.10 and the highest posterior density interval at 95% of probability (HPD_95%_) were also estimated. Analyses were performed using the TM software [25].

## 3. Results

Descriptive statistics for the ADG between −14 and 28 DPV are displayed in Table 1. Overall, pigs from the C group showed a slightly greater ADG than challenged pigs except for batch 1. The ADG (coefficient of variation; %) ranged from 594.3 (24.9) to 756.2 (10.6) g/day for the E group and from 556.2 (21.5) to 794.7 (4.9) g/day for the C group. The lowest ADG were observed in batches 1, 2, and 5. The HP concentration at 4 DPV was variable between and within batches ranging from 0.70 (70.5) to 1.95 (42) mg/mL for the E group and from 1.09 (54.7) to 2.07 (33.1) mg/mL for the C group (Table 1). The highest HP concentrations at 4 DPV were reported in batches 2, 3, and 5. The coefficients of variation for ADG and HP were high, indicating a considerable variability between animals, and in general, higher in the E group than in the C group.

### 3.1. Descriptive Statistics for the Novel Resilience Indicators

Descriptive statistics of the suggested resilience indicators are given in Table 2. Average ∆BW and %BW were −0.68 kg and −1.42%, respectively, indicating that on average, the observed BW of challenged pigs at 28 DPV was lower than the expected BW given the theoretical growth curve. Average ∆HP and %HP were +0.03 mg/mL and +5.40%, respectively, showing an increment of HP concentration in plasma at 4 DPV. All the resilience indicators had high SD values.

Phenotypic correlations between the resilience indicators are reported in Table 3. A negative and low correlation was reported between ∆BW and ∆HP (r = −0.09, *p* < 0.05), suggesting that they capture different aspects of resilience. ∆BW and ∆BW_ADG_ were moderately correlated (r = 0.52, *p* < 0.001).

### 3.2. Classification of Pigs’ Resilience

**Figure 2:**
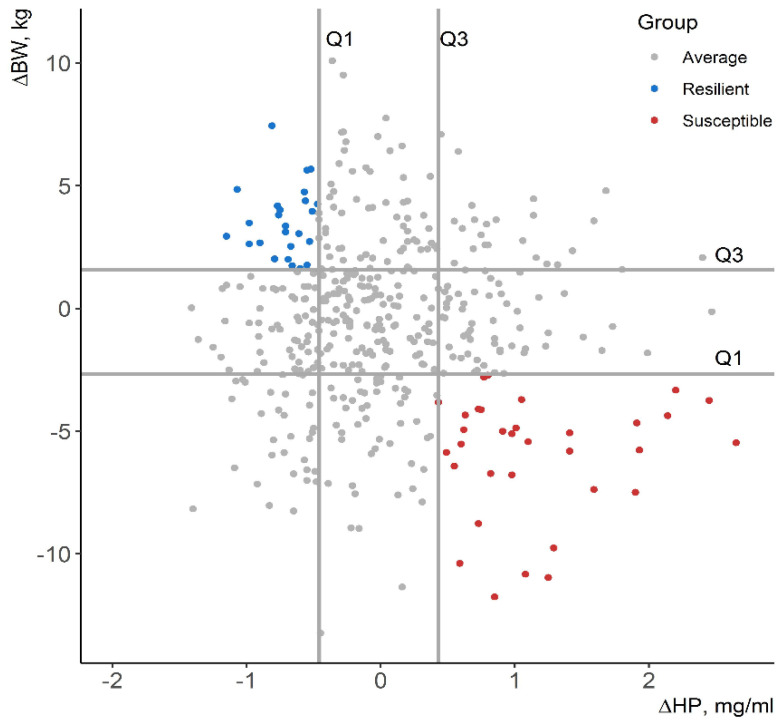
Classification of pigs as resilient, average, and susceptible based on the first (Q_1_) and the third (Q_3_) quartiles of ∆BW and ∆HP. ∆BW: body weight deviation from the expected growth curve of control pigs at 28 days post-vaccination, ∆HP: haptoglobin increment at 4 days post-vaccination.

Pigs were classified based on the resilience indicated by both the deviation from the expected growth curve and the increment of HP at 4 DPV. First, individuals were grouped into R or S groups based on ∆BW and ∆HP (Figure 2). On average, the resilient pigs (N = 25) showed positive values of ∆BW (+3.54 kg) and %BW (+6.60%) and negative values of ∆HP (−0.71 mg/mL) and %HP (−61.2%). In contrast, the susceptible group (N = 33) had low and negative values of ∆BW (−6.00 kg) and %BW (−11.7%) and positive values of ∆HP (+1.17 mg/mL) and %HP (+108%) (Table 4).

**Figure 3:**
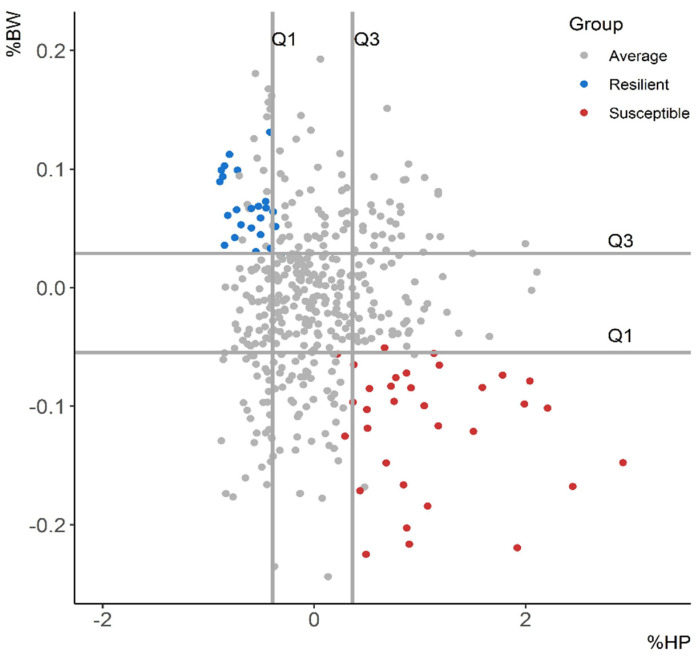
Projection of the resilient, average, and susceptible groups obtained with the first (Q_1_) and third (Q_3_) quartiles of ∆BW and ∆HP on the plane defined by %HP and %BW. Individuals were colored according to their group classification using the criterion from Figure 2 to visualize concordance between both methods. ∆BW: body weight deviation from the expected growth curve of control pigs at 28 days post-vaccination (DPV), ∆HP: haptoglobin increment at 4 DPV, %BW: ratio between ∆BW and the expected body weight at 28 DPV, %HP: ratio between ∆HP and the basal level of haptoglobin.

Pigs were also grouped into R and S groups based on the resilience indicated by %BW and %HP (Figure 3). Individuals were colored according to their group classification using ∆BW and ∆HP in order to visualize the concordance between the first and the second classifications. The concordance was high with a kappa value of 0.8 and an overall agreement of 95%. Pigs were also classified into R, A, and S based on the observed BW_28_ (Figure S1) and the combination of ∆BW_ADG_ and ∆HP (Figure S2). The concordance was low (kappa = 0.1) between the classification obtained by the observed BW_28_ and the combination of “∆BW and ∆HP” and moderate (kappa = 0.5) between the classification indicated by “∆BW_ADG_ and ∆HP” and “∆BW and ∆HP”.

**Figure 4:**
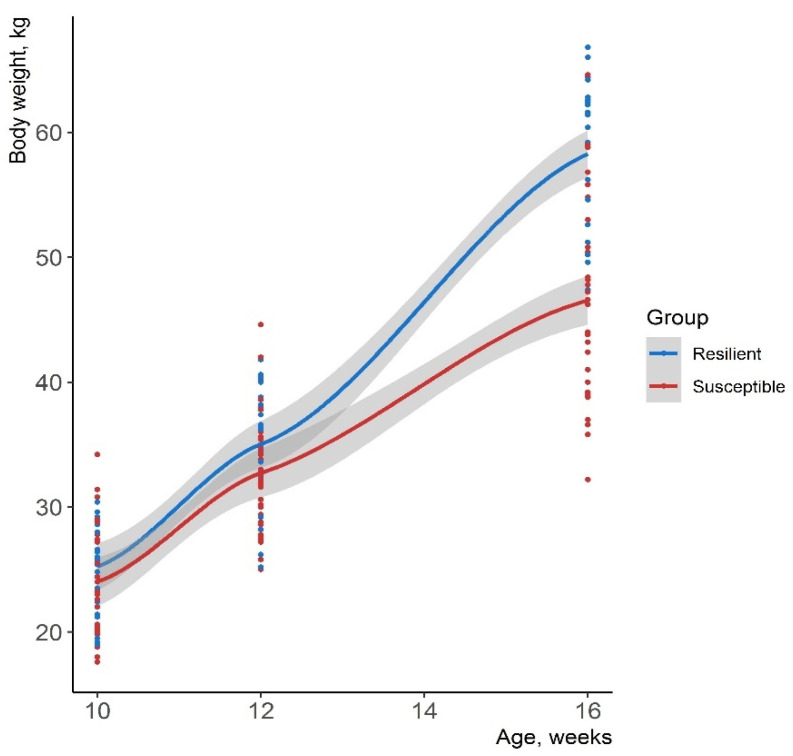
Growth curves of pigs from the resilient and susceptible groups. The grey band represents the confidence interval. Individuals were colored according to their group classification using the criterion from Figure 2.

The growth curves of animals from the R and S groups were similar at the beginning of the experiment (Figure 4). After the challenge at 12 weeks of age, resilient animals were able to withstand the perturbations and showed a faster growth than susceptible ones. At the end of the fattening period (30 weeks of age), the resilient pigs showed a greater carcass weight than susceptible ones (107.7 and 92.1 kg, respectively).

### 3.3. Heritability Estimates

The features of the marginal posterior distributions of the heritability estimates are displayed in Table 5. Both ∆BW and %BW had a moderate heritability of 0.33 and 0.37, with P_0.10_ of 0.94 and 0.93, respectively. Heritability estimates of ∆HP and %HP were 0.16 and 0.13, with P_0.10_ of 0.66 and 0.53, respectively.

## 4. Discussion

There is no consensus on the definition of resilience nor on how to measure it. Selection for animals with a natural ability to cope with stressors and adapt to sudden changes has been proposed as a contribution to the sustainability of animal breeding [26]. However, in order to select these animals, we first need to be able to measure the trait. Different productivity and non-productivity indicators that reflect the disturbance caused by stressors have been used as indicators of resilience in several livestock species. Bai et al. [23] used quartiles of growth and the treatment rate in response to an infectious challenge to quantify the pigs’ resilience and considered animals with low number of treatments received and high growth rate as resilient. Other authors have defined resilience as the ability of animals to be minimally affected by perturbations and proposed resilience indicators based on the deviation of production traits such as the variance of deviations, the residual variance, and the skewness of deviations, considering individuals with the highest deviations as the most susceptible ones [5,6,8]. In the present work, we suggest novel resilience indicators based on the deviation from the expected BW given the growth curve of control pigs (∆BW) and the increment of the acute-phase protein HP at 4 DPV (∆HP), as a stress indicator. Animals that were able to maintain their productivity (positive values of ∆BW) and had a minor activation of HP (lower values of ∆HP) were deemed resilient.

It is important to study the vaccine response since commercial animals are routinely vaccinated. The response to an attenuated Aujeszky vaccine was used to identify pigs with increased resilience. The vaccine response is an attractive alternative to natural infection because all animals can be vaccinated at the same dose, age, and time and, thus, response phenotypes can be collected with higher consistency and accuracy. This strategy has been previously used to study the genetic variability of the immune response to Aujeszky disease [27], influenza virus [28], European and American PRRS virus [29], and atrophic rhinitis [30]. Another advantage of using vaccines as a proxy of pathogen infection is that vaccinated and non-vaccinated animals can be reared in the same batch under the same environmental conditions, eliminating the confusion between treatment and batch.

Vaccinated and control pigs were reared in five different batches where additional uncontrolled events occurred during the experiment and affected both E and C groups. In addition to the vaccine challenge, the first batch suffered from metabolic stress due to a failure in feed distribution, while batches 2 and 5 were exposed to an additional stress due to disease (gastric ulcers outbreak and respiratory disease). As a consequence, these batches had the lowest ADG. These results were expected since affected animals have a reduced feed intake and redirect energy to fight against diseases, and therefore, they convert food less efficiently into edible products [31]. On the other hand, batches 2, 3, and 5 had the highest HP concentration at 4 DPV, indicating that, in addition to the vaccine challenge, the batches were exposed to different stressful conditions. The HP concentrations after the challenge were lower than those in the literature for Aujeszky disease [21,22]. Carpintero et al. [22] and Parra et al. [21] reported greater average HP responses (3.96 and 1.81 mg/mL, respectively). One possible explanation could be that the animals were challenged with the Aujeszky disease vaccine three and two times, respectively, whereas, in our case, pigs were challenged with one dose of the attenuated Aujeszky disease vaccine.

Vaccinated pigs had a reduced growth compared to control pigs. On average, the observed BW_28_ of challenged pigs was lower than expected. Besides, the HP concentration was 5% higher at 4 DPV. Among the 445 challenged pigs, 25 were deemed resilient and 33 were deemed susceptible. The observed BW_28_ for the resilient pigs was 6.6% (+3.45 kg) higher than expected, while the observed BW_28_ for the susceptible pigs was 11.7% (−6 kg) lower than expected. Regarding the HP response, the HP concentration highly increased after the challenge in the S group. Thus, our approach allowed us to separate two groups with extreme responses to challenge. Resilience was also assessed based on the combination of %BW and %HP. The concordance was high between both methods, indicating that ∆BW and ∆HP are not sensitive to the animal’s expected BW nor the basal level of HP and are consequently potential indicators of resilience. Moreover, the concordance was low between the classifications obtained by the combination of “∆BW and ∆HP” and the observed BW_28_, indicating that ∆BW and ∆HP do not only capture the differences in the observed BW. Finally, the concordance was moderate between the resilience classification indicated by the combination of “∆BW and ∆HP” and “∆BW_ADG_ and ∆HP”. Thus, pigs could be consistently classified into R, A, and S based on ∆BW_ADG_ and ∆HP without using a control group in the same batch of animals. The ∆BW_ADG_ indicator would enable the application of the proposed resilience criteria under practical conditions where it is not possible or desirable to have non-vaccinated animals such as in the selection of nucleus farms.

Since there is no consensus on the quantification method of resilience, completely different approaches may be applied depending on the resilience definition and the objectives of the study or breeding program. In this work, resilience was studied as a qualitative trait (R, A and S) and the classification of the pigs’ resilience was based on the quartiles of ∆BW and ∆HP as suggested by Bai et al. [23]. Nevertheless, resilience was also analyzed as a quantitative trait (R=α∗ΔBW+β∗ΔHP) with our database and similar results were obtained compared with the qualitative ones (Supplementary Materials), suggesting that our qualitative approach is robust enough to set up resilience criteria.

The heritability estimates of the resilience indicators were moderate for ∆BW and %BW and low-to-moderate for ∆HP and %HP. Heritabilities for ∆BW at 28 DPV have not been reported before, but our estimated value is similar to the heritability of BW reported in Duroc pigs at 180 days of age (0.31) [32]. Our heritability estimates for HP are within the range of those reported in non-specific pathogen-free pig farms (0.20) [33] and in growing pigs (0.14) [34]. Noteworthy, the experimental sample size limits the accuracy of the heritability estimates. However, P_0.10_ (i.e., the probability of the heritability being greater than 0.10) showed that the resilience indicators are genetically controlled and consequently, may be improved through selective breeding but the genetic correlations of these resilience indicators will be robustly established in follow-up studies, with a bigger number of animals, before including them in the breeding selection program.

## 5. Conclusions

Altogether, we propose ∆BW and ∆HP as novel resilience indicators in growing pigs. The suggested indicators are easy to measure, genetically controlled, and show substantial variability between animals. Thus, they may be improved through selective breeding. This approach may be applied to quantify resilience in other species using different infectious and non-infectious challenges. Moreover, genomic studies on R and S animals can help in elucidating the molecular basis of the different responses in R and S animals. This work is a starting point of the study of the resilience in pigs. Further analyses are needed to validate our findings.

## Figures and Tables

**Table 1:** Mean (coefficient of variation; %) for the average daily gain (ADG; g/day) between −14 and 28 days post-vaccination (DPV) and the serum haptoglobin concentration (HP; mg/mL) at 4 DPV.

Group	Experimental Group			Control Group		
Batch	N	ADG	HP	N	ADG	HP
1	84	594.3 (24.9)	0.70 (70.5)	20	556.2 (21.5)	1.09 (54.7)
2	95	624.8 (17.0)	1.48 (58.0)	16	707.7 (14.1)	1.59 (31.9)
3	86	756.2 (10.6)	1.95 (42.0)	21	768.4 (10.7)	2.07 (33.1)
4	86	756.0 (11.9)	1.08 (55.3)	23	794.7 (4.9)	1.07 (53.5)
5	94	662.4 (16.7)	1.40 (37.3)	15	749.3 (16.2)	1.30 (39.3)
All batches	445	677.7 (18.8)	1.33 (59.4)	95	716.9 (17.8)	1.42 (49.1)

**Table 2:** Descriptive statistics for the resilience indicators in pigs from the experimental group.

Trait	Mean	SD ^1^	Min	Max
∆BW ^2^ (kg)	−0.68	3.64	−13.2	+10.1
%BW ^3^ (%)	−1.42	7.26	−24.4	+19.3
∆HP ^4^ (mg/mL)	+0.03	0.70	−1.41	+2.65
%HP ^5^ (%)	+5.40	60.4	−89.2	+292

^1^ Standard deviation; ^2^ Body weight deviation from the expected growth curve of control pigs at 28 days post-vaccination (DPV); ^3^ Ratio between ∆BW and the expected body weight at 28 DPV given the growth curve of control pigs; ^4^ Haptoglobin increment at 4 DPV; ^5^ Ratio between ∆HP and the basal level of haptoglobin.

**Table 3:** Correlations between the resilience indicators.

Trait	∆BW ^1^	∆HP ^2^	%BW ^3^	%HP ^4^	∆BW_ADG_^5^
∆BW	1	−0.09 *	0.99 ***	−0.14 **	0.52 ***
∆HP		1	−0.09 ^ns^	0.94 ***	−0.02 ^ns^
%BW			1	−0.14 **	0.51 ***
%HP				1	−0.05 ^ns^

^1^ Body weight deviation from the expected growth curve of non-vaccinated pigs at 28 days post-vaccination (DPV); ^2^ Haptoglobin increment at 4 DPV; ^3^ Ratio between ∆BW and the expected body weight at 28 DPV given the growth curve of control pigs. ^4^ Ratio between ∆HP and the basal level of haptoglobin; ^5^ Body weight deviation from the expected BW at 28 DPV estimated based on each pig’s average daily before challenge. * *p* < 0.05; ** *p* < 0.01; *** *p* < 0.001; ^ns^ non-significant.

**Table 4:** Mean and standard deviation (SD) of the resilience indicators of pigs from the resilient (R, N = 25) and susceptible (S, N = 33) groups.

Trait	Group	Mean	SD
∆BW ^1^ (kg)	R	+3.54	1.42
	S	−6.00	2.44
%BW ^2^ (%)	R	+6.60	2.81
	S	−11.7	5.13
∆HP ^3^ (mg/mL)	R	−0.71	0.18
	S	+1.17	0.61
%HP ^4^ (%)	R	−61.2	18.9
	S	+108.4	68.2

^1^ Body weight deviation from the expected growth curve of control pigs at 28 days post-vaccination (DPV); ^2^ Ratio between ∆BW and the expected body weight at 28 DPV given the growth curve of control pigs; ^3^ Haptoglobin increment at 4 DPV; ^4^ Ratio between ∆HP and the basal level of haptoglobin.

**Table 5:** Heritability estimates for the resilience indicators.

Trait	Mean ^1^	P_0.10_^2^	HPD_95%_^3^
∆BW ^4^	0.33	0.94	0.02–0.65
%BW ^5^	0.37	0.93	0.02–0.74
∆HP ^6^	0.16	0.66	0.00–0.38
%HP ^7^	0.13	0.53	0.00–0.32

^1^ Mean of the marginal posterior distribution of the heritability; ^2^ Probability of the heritability estimate being greater than 0.10; ^3^ Highest posterior density interval at 95% of probability; ^4^ Body weight deviation from the expected growth curve of non-vaccinated pigs at 28 days post-vaccination (DPV); ^5^ Ratio between ∆BW and the expected body weight at 28 DPV given the growth curve of control pigs; ^6^ Haptoglobin increment at 4 DPV; ^7^ Ratio between ∆HP and the basal level of haptoglobin.

## Data Availability

The data presented in this study are available on request from the corresponding author.

## Supplementary Materials

The following are available online at https://www.mdpi.com/article/10.3390/ani11102970/s1. Figure S1. Projection of the resilient, average and susceptible groups obtained with the first (Q_1_) and third (Q_3_) quartiles of ∆BW and ∆HP on the plane defined by the first (Q_1_) and third (Q_3_) quartiles of BW at 28 days post-vaccination (DPV). Individuals were colored according to their group classification using the criterion from Figure 2 to visualize concordance between both methods. ∆BW: body weight deviation from the expected growth curve of control pigs at 28 DPV, ∆HP: haptoglobin increment at 4 DPV. Figure S2. Projection of the resilient, average and susceptible groups obtained with the first (Q_1_) and third (Q_3_) quartiles of ∆BW and ∆HP on the plane defined by ∆HP and ΔBW_ADG_. Individuals were colored according to their group classification using the criterion from Figure 2 to visualize concordance between both methods. ∆BW: body weight deviation from the expected growth curve of control pigs at 28 days post-vaccination (DPV), ∆HP: haptoglobin increment at 4 DPV, ΔBW_ADG_: Body weight deviation from the expected BW at 28 DPV estimated based on each pig’s average daily before challenge. Figure S3. Distribution of the quantitative resilience indices Table S1. Correlations between the resilience indicators based on quantitative indices.
