# Supplementary material for: A Methodology to Quantify Resilience in Growing Pigs
Source: Animals (Basel). 2021 Oct 15;11(10):2970. doi: 10.3390/ani11102970 (PMC8532637; doi:10.3390/ani11102970)
Supplement: Supplementary file 1 [file animals-11-02970-s001.zip › animals-1390582-supplementary.pdf]

Supplementary material

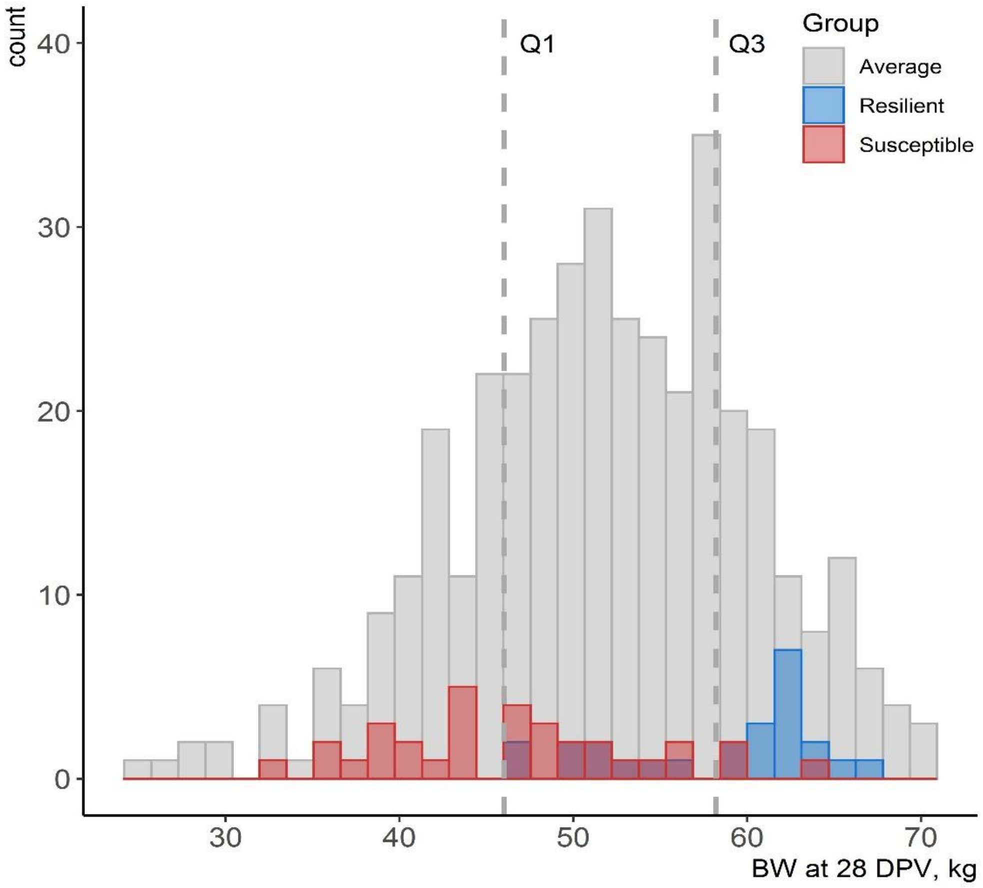

Figure S1. Projection of the resilient, average and susceptible groups obtained with the first (Q1) and third (Q3) quartiles of ΔBW and ΔHP on the plane defined by first (Q1) and third (Q3) quartiles of BW at 28 days post-vaccination (DPV). Individuals were coloured according to their group classification using the criterion from Figure 2 to visualize concordance between both methods. ΔBW: body weight deviation from the expected growth curve of control pigs at 28 DPV, ΔHP: haptoglobin increment at 4 DPV.

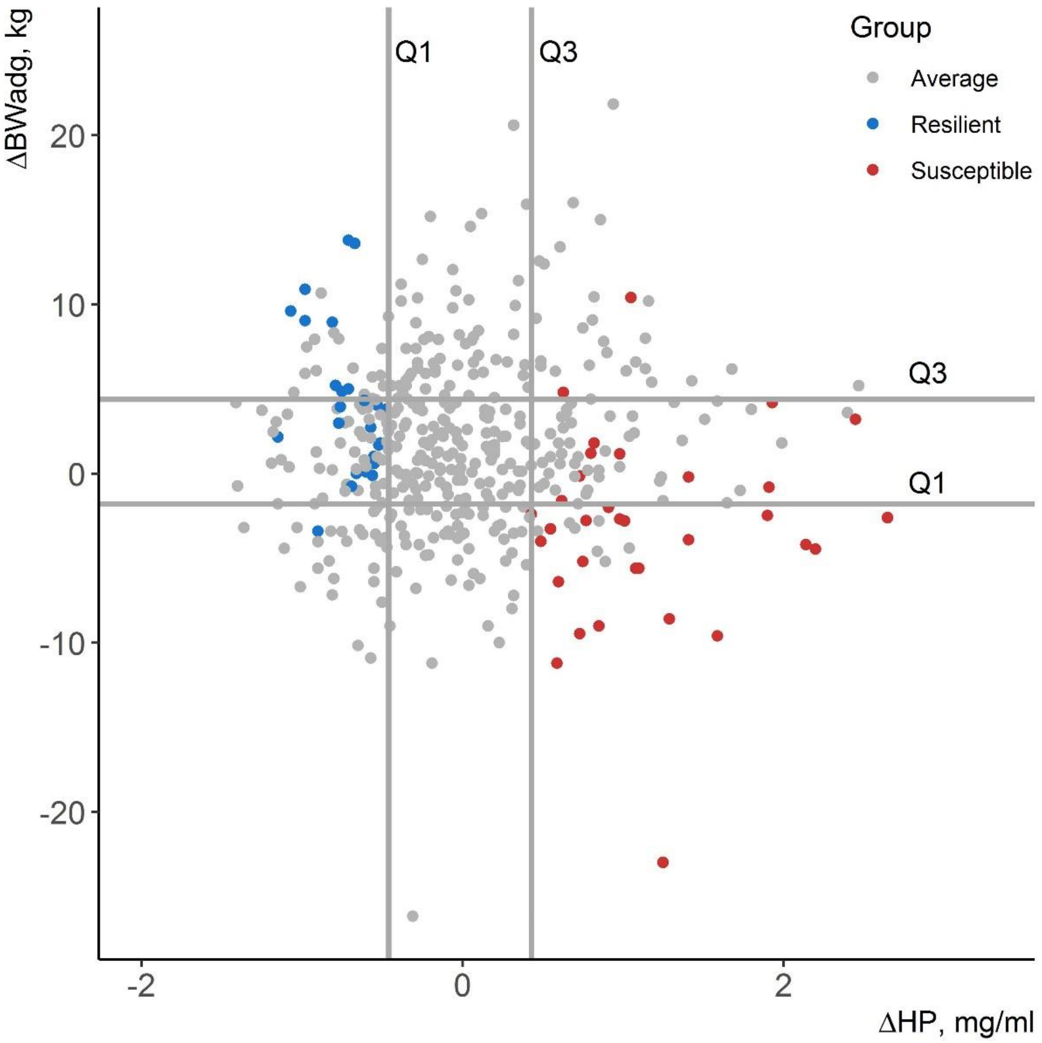

Figure S2. Projection of the resilient, average and susceptible groups obtained with the first (Q1) and third (Q3) quartiles of ΔBW and ΔHP on the plane defined by ΔHP and ΔBW_{ADG}. Individuals were colored according to their group classification using the criterion from Figure 2 to visualize concordance between both methods. ΔBW: body weight deviation from the expected growth curve of control pigs at 28 days post-vaccination (DPV), ΔHP: haptoglobin increment at 4 DPV, ΔBW_{ADG}: Body weight deviation from the expected BW at 28 DPV estimated based on each pig's average daily before challenge

Resilience as a quantitative trait

Resilience was defined as the ability of pigs to withstand stressors and maintain expected growth. A total of 445 commercial Duroc pigs were challenged with an attenuated Aujeszky vaccine at 12 weeks of age (85.6 ± 2.4 days of age) and 95 control pigs were inoculated with phosphate-buffered saline. Pigs were weighed at -14 (BW_{-14}), 0 (BW_0) and 28 (BW_{28}) days post-vaccination (DPV) and bled at 0 and 4 DPV. The acute-phase protein haptoglobin (HP) was quantified at 0 and 4 DPV. Body weight (BW) and HP data were analysed to quantify the pigs' resilience. In the paper, resilience was studied as a qualitative trait (resilient, average and susceptible) and the BW deviation (ΔBW) from the expected growth curve of control pigs and the increment (ΔHP) of HP at 4 DPV were suggested as potential resilience indicators. Herein, resilience was also evaluated as a quantitative trait. Data were standardised and the following indices were defined to examine ΔBW and ΔHP.

$$R_1 = 0.5 * \Delta BW - 0.5 * \Delta HP$$

$$R_2 = 0.5 * \%BW - 0.5 * \%HP$$

$$R_3 = 0.5 * \Delta BW_{ADG} - 0.5 * \Delta HP$$

$$R_4 = BW_{28}$$

where ΔBW and ΔHP are as described previously, %BW is the ratio between ΔBW and the expected BW_{28} given the growth curve on control pigs, %HP is the ratio between ΔHP and the basal level of HP and ΔBW_{ADG} is the BW deviation from the expected BW_{28} estimated based on each pig's average daily gain before the vaccine challenge. The distribution of the different indices is displayed in Figure S3.

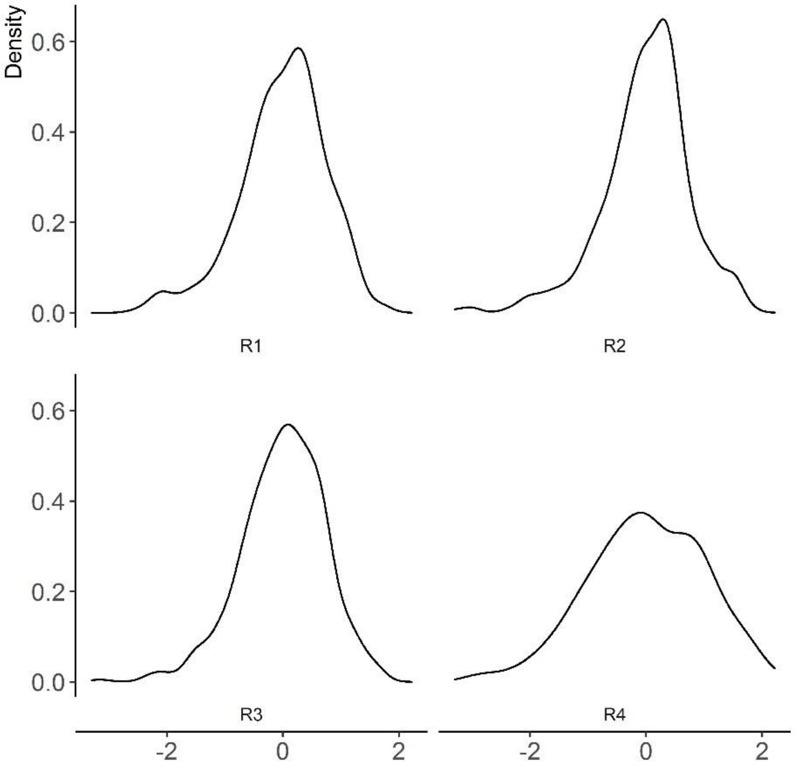

Figure S3. Distribution of the quantitative resilience indices.

Correlations were estimated in order to evaluate the relationship between the different indices (Table S1). A strong and positive correlation (0.97) was obtained between R_1 and R_2, indicating that they reflect the same aspect of resilience and thus the resilience criterion based on ΔBW and ΔHP is not sensitive to the animal's BW nor the basal level of HP. R_1 and R_3 were positively correlated (0.77), suggesting that in practice pigs could be classified based on ΔBW_{ADG} and ΔHP without using the control group. Finally, the correlation was low between R_1 and R_4, indicating that R_1 reflect more than the differences in the animals' BW at 28 DPV. These findings are in line with our previous results and corroborate the robustness of ΔBW and ΔHP (R_1) as resilience indicators in pigs.

Table S1. Correlations between the resilience indices based on quantitative indices.

	$\mathbf{R_2}$	$\mathbf{R_3}$	$\mathbf{R_4}$
$\mathbf{R_1}$	0.97***	0.77***	0.33***
$\mathbf{R_2}$	-	0.73***	0.36***
$\mathbf{R_3}$		-	ns

***$p < 0.001$, ns: non-significant.
